# p21 facilitates chronic lung inflammation via epithelial and endothelial cells

**DOI:** 10.18632/aging.204622

**Published:** 2023-03-30

**Authors:** Naama Levi, Nurit Papismadov, Julia Majewska, Lior Roitman, Noa Wigoda, Raya Eilam, Michael Tsoory, Ron Rotkopf, Yossi Ovadya, Hagay Akiva, Ofer Regev, Valery Krizhanovsky

**Affiliations:** 1 Department of Molecular Cell Biology, The Weizmann Institute of Science, Rehovot 7610001, Israel; 2 Department of Life Sciences Core Facilities, The Weizmann Institute of Science, Rehovot 7610001, Israel; 3 Department of Veterinary Resources, The Weizmann Institute of Science, Rehovot 7610001, Israel; 4 Department of Immunology, The Weizmann Institute of Science, Rehovot 7610001, Israel

**Keywords:** cellular senescence, chronic lung inflammation, p21 (CDKN1A)

## Abstract

Cellular senescence is a stable state of cell cycle arrest that regulates tissue integrity and protects the organism from tumorigenesis. However, the accumulation of senescent cells during aging contributes to age-related pathologies. One such pathology is chronic lung inflammation. p21 (CDKN1A) regulates cellular senescence via inhibition of cyclin-dependent kinases (CDKs). However, its role in chronic lung inflammation and functional impact on chronic lung disease, where senescent cells accumulate, is less understood. To elucidate the role of p21 in chronic lung inflammation, we subjected p21 knockout (*p21^-/-^*) mice to repetitive inhalations of lipopolysaccharide (LPS), an exposure that leads to chronic bronchitis and accumulation of senescent cells. p21 knockout led to a reduced presence of senescent cells, alleviated the pathological manifestations of chronic lung inflammation, and improved the fitness of the mice. The expression profiling of the lung cells revealed that resident epithelial and endothelial cells, but not immune cells, play a significant role in mediating the p21-dependent inflammatory response following chronic LPS exposure. Our results implicate p21 as a critical regulator of chronic bronchitis and a driver of chronic airway inflammation and lung destruction.

## INTRODUCTION

Chronic lung inflammation is the major component of chronic obstructive pulmonary disease (COPD), the third most common cause of death in developed countries, that has become a global epidemic and is increasing worldwide [1, 2]. This disease is associated with chronic inflammation of the peripheral airways and lung parenchyma (chronic bronchitis), the subsequent destruction of the lung alveoli (emphysema), and the accumulation of senescent cells in different lung compartments [1, 3–6]. The presence of senescent cells is associated with chronic inflammation, which is characterized by the recruitment of innate and adaptive immune cells, [7–10]. The peripheral immune cells are attracted to the lung tissue by secretion of pro-inflammatory mediators, including cytokines, chemokines and growth factors, by epithelial [11], endothelial [12] and fibroblast [13] cells in the lungs. Epithelial cells are activated in response to chronic airway irritation, caused by smoke and other pollutants, to produce inflammatory mediators [11, 14–16]. Endothelial cells are also activated following chronic damage to the lung [12, 17] and express cytokines and chemokines, via NF-κB transcriptional activation [18]. Activated endothelial cells also express adhesion molecules which increase vascular barrier permeability, such as intercellular adhesion molecule-1 (ICAM-1), and vascular cell adhesion molecule-1 (VCAM-1), which promotes adherence of immune cells [19–21]. However, the contribution of specific molecular and cellular mechanisms in epithelial and endothelial cells to the chronic inflammation requires further investigation.

Aging is associated with an increased risk of chronic inflammatory lung diseases [22–24]. One of the hallmarks of aging is cellular senescence, and indeed, senescent cells of epithelial, endothelial, fibroblast and immune origin accumulate in lungs with chronic inflammation [25, 26]. Cellular senescence refers to a stable state of cell cycle arrest, accompanied by a profound secretory phenotype [27–29]. On the short term, the presence of senescence cells plays a role in tumor suppression, wound healing, as well as in embryonic development [30–36]. Conversely, accumulation of senescent cells in tissues, a process that occurs during aging, stimulates tumorigenesis and the development of age-related diseases by promoting chronic inflammation [37–39]. This process is mediated by expression and secretion of proteins, members of the senescence associated secretory phenotype (SASP), which leads to chronic activation of immune responses in the lungs [40–45]. However, the relative contribution of senescence mechanisms in different cell types to the chronic inflammation is not sufficiently understood.

The senescence growth arrest is established and maintained by the p53-p21 and p16-Rb pathways [27, 46–48]. One of the cell-cycle inhibitors that is often expressed by senescent cells is the cyclin-dependent kinase inhibitor (CDKI) p21 (also termed CDKN1A). p21 can inhibit the cyclin-dependent kinases (CDKs) CDK2 and CDK4, leading to suppression of retinoblastoma protein (Rb) phosphorylation, and to a subsequent downregulation of genes necessary for cell-cycle progression [42, 49–51]. In addition to its role in senescence, p21 is involved in lung pathologies and p21 ablation attenuates the chronic inflammatory responses and emphysema [52–55]. The contribution of p21 to chronic lung inflammation may rely on its ability to regulate multiple phenotypes of senescent cells. Indeed, one of the characteristics of senescent cells that is necessary for their persistence, resistance to cell death [56, 57] is mediated by p21 and BCL-2 family members [58–60].

While p21 is a key regulator of cellular senescence, its role in specific cell populations in the lung during chronic inflammation and functional output of chronic lung disease requires further investigation. Of note, p53, the upstream mediator of p21 expression, facilitates chronic lung inflammation in bronchial epithelial cells by promoting cellular senescence [26]. We, therefore, utilized a LPS inhalation-induced chronic bronchitis procedure [26] to study the effects of repetitive LPS exposure on p21 knockout (*p21^-/-^*) mice. In this model, p21 knockout leads to a decrease in the inflammatory response, reduced accumulation of senescent cells, improved fitness, and alleviation of the pathological manifestations of chronic lung inflammation. Furthermore, we aimed to examine the specific contribution of the epithelial, endothelial and immune compartments to chronic bronchitis pathology. We revealed that resident epithelial and endothelial cells in the lungs, but not immune cells, play a significant role in mediating the p21-dependent inflammatory response following chronic LPS exposure. Overall, these findings suggest that p21 is a crucial regulator of chronic airway inflammation and lung destruction.

## RESULTS

### Chronic LPS inhalation induces p21-dependent cellular senescence in bronchial epithelia

**Figure 1:**
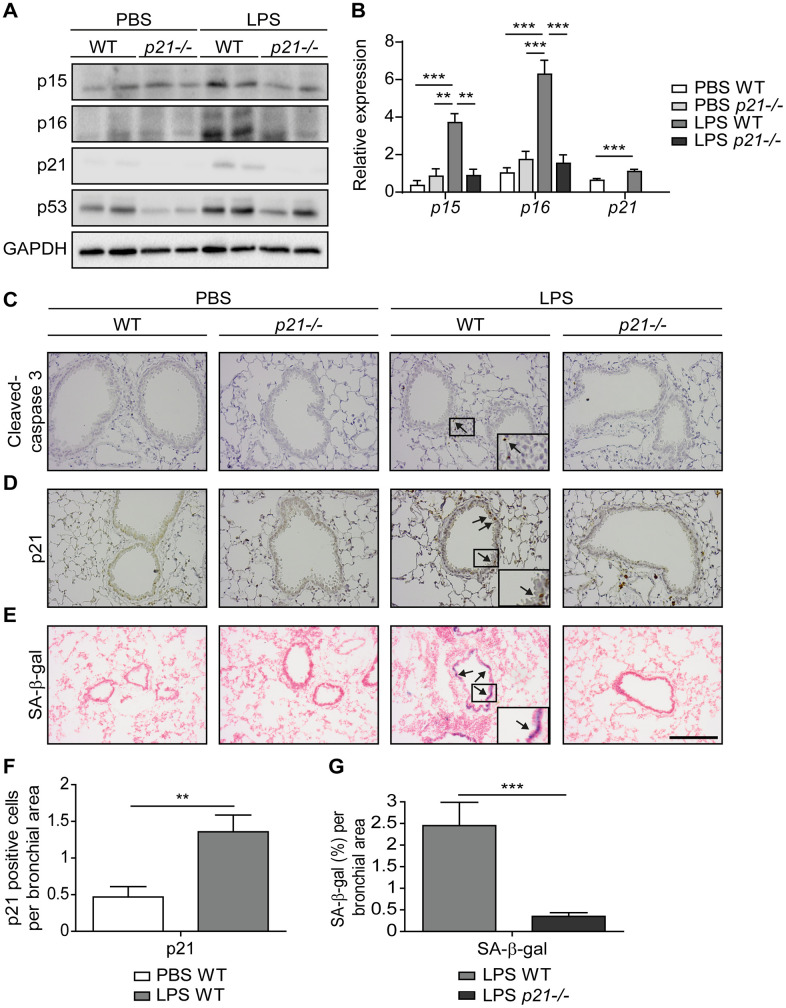
**Accumulation of senescent cells is decreased in the lungs of *p21^-/-^* mice.** WT and *p21^-/-^* mice were exposed to either PBS or aerosolized LPS (0.5 mg/ml), 3 times a week for 10 weeks. At 48 hours following the last LPS exposure, the lungs were harvested and frozen. Alternatively, lungs were harvested, fixed, and analyzed for markers of senescence. (**A**) Representative immunoblots for senescence-associated proteins p15, p16, p21 and p53 in the mice lungs. (**B**) mRNA expression levels of senescence markers *p15*, *p16* and *p21* in the mice lungs. (**C**, **D**) Immunohistochemistry (IHC) of lung sections for cleaved caspase 3, (**C**) and p21, (**D**). Scale bar represents 200μm. (**E**) SA-β-Gal staining of lung sections. Scale bar represents 200μm. (**F**) Quantification of the number of p21 positive cells per bronchial area, of the lung sections presented in (**D**). (**G**) Quantification of SA-β-gal (%) per bronchial area, of the lung sections presented in (**E**). Data information: Data were analyzed using one-way ANOVA, *P<0.05. **<0.005. ***P<0.0005 (**B**), and by Student’s t-test, *p<0.05, **p<0.01, and ***p<0.005 (**F**, **G**). Data represent mean ±SEM (**A**, n=3; **B**, n=3-10; **C**–**G**, n=3-6 independent repeats).

Repetitive lung injury causes p53-dependent accumulation of senescent cells in the lungs, which contributes to chronic lung inflammation and promotes chronic bronchitis [26]. p21, a downstream mediator of p53, regulates cell cycle arrest and the viability of senescent cells [60]. Here we set to understand the effect of p21 during chronic lung inflammation. To this end, we exposed wild type (WT) and p21 knockout (*p21^-/-^*) 8-week-old female mice to chronic LPS-inhalation (0.5 mg/ml) regimen, which causes chronic lung inflammation [26], or to PBS-inhalation regimen as a control, 3 times a week for 10 weeks. In order to understand the effect of p21 knockout on accumulation of senescent cells following exposure to repetitive LPS inhalations, we analyzed the mice lungs for cellular senescence markers. We first evaluated the protein expression levels of the senescence markers p15, p16, p21 and p53 in the lungs of WT and *p21^-/-^* mice by immunoblot analysis and the mRNA expression levels of *p15*, *p16* and *p21*. This analysis revealed increased expression of the senescence markers in the lungs of LPS exposed WT mice, compared to PBS exposed mice, while p21 knockout abolished this effect (Figure 1A, 1B and Supplementary Figure 1A, representative immunoblots).

Senescent cells exacerbate chronic bronchitis pathology and their elimination limits the disease progression [26]. Furthermore, p21 silencing results in elimination of senescent cells via apoptosis [60]. To determine the effect of p21 knockout on accumulation of senescent bronchial epithelial cells we evaluated lung sections stained for the apoptotic marker cleaved caspase-3, a cellular senescence marker p21, and for SA-β-gal activity (Figure 1C–1E). Neither WT nor *p21^-/-^* mice presented caspase-3 cleavage following chronic LPS exposure (Figure 1C and Supplementary Figure 1B - positive control), suggesting that the bronchial epithelial cells do not undergo significant apoptosis in our experimental end-point. The immunoblot analysis of the cleavage caspase-3 did not reveal a significant difference in expression of this marker between the genotypes (Supplementary Figure 1C). Alternatively, immunohistochemical analysis of p21 expression revealed a marked elevation in the amount of p21 expressing cells in LPS exposed WT mice, compared to the PBS exposed mice (Figure 1D, 1F). Furthermore, the levels of SA-β-gal activity in the lungs of LPS exposed WT mice were increased relative to lungs of PBS exposed mice (Figure 1E), while p21 knockout abolished this effect (Figure 1E, 1G). These results suggest that following chronic LPS exposure lung cells undergo senescence via a p21-dependent pathway.

### *p21^-/-^* diminishes the pathological manifestations of chronic bronchitis

**Figure 2:**
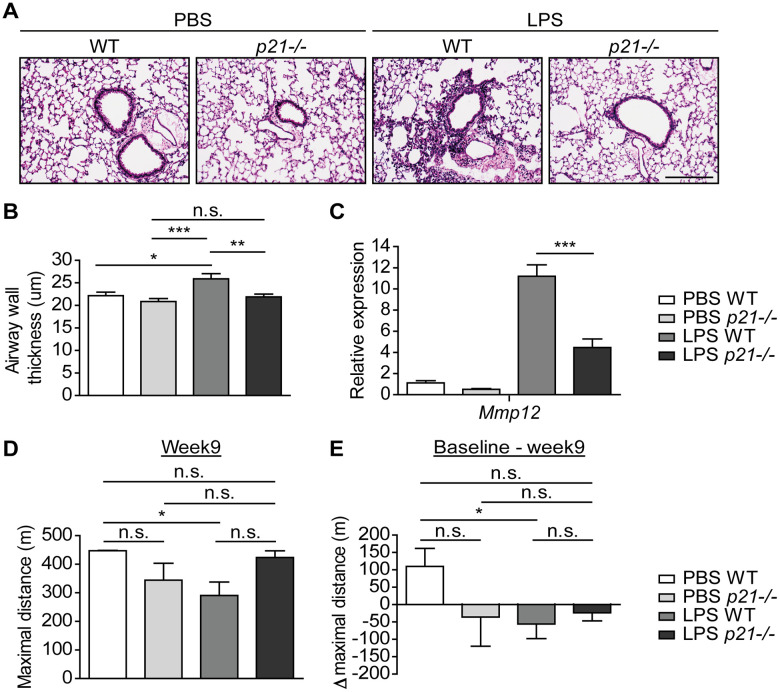
***p21^-/-^* improves the pathophysiology triggered by chronic LPS exposure.** WT and *p21^-/-^* mice were exposed to either PBS or aerosolized LPS (0.5 mg/ml), 3 times a week for 10 weeks. At 48 hours following the last LPS exposure the lungs were harvested, fixed, and stained by H&E. (**A**) Representative images of lung sections featuring bronchi and alveoli of WT and *p21^-/-^* mice. Scale bar represents: 200μm. (**B**) Airway wall thickness of the lung described in (**A**). (**C**) mRNA expression levels of chronic bronchitis associated component *Mmp12* in the lungs described in (**A**). (**D**) At week 9, mice fitness was assessed by a treadmill assay and was presented as maximal actual distance. (**E**) Delta maximal actual distance was calculated from the baseline point until week 9 of the mice described in (**D**). Data information: Data were analyzed using one-way ANOVA, *P<0.05. **<0.005. ***P<0.0005 (**B**, **C**), and by Mann Whitney u test (**D**, **E**), *p<0.05. Data represent mean ±SEM (**A**, **B**, n=5; **C**, n=3-8; **D**, **E**, n=4-5 independent repeats).

In order to understand the effect of p21 knockout on the progression of chronic bronchitis following chronic LPS exposure, we evaluated the pathology parameters of the mice in our experimental groups. Hematoxylin and eosin (H&E) staining revealed that chronic LPS exposure in WT mice resulted in airway inflammation, as reflected by infiltration of immune cells around the bronchi (Figure 2A), and in a significant increase in bronchial wall thickness, compared to PBS treated mice (Figure 2B). Strikingly, *p21^-/-^* mice had not shown accumulation of immune cells around the bronchi following chronic LPS exposure (Figure 2A) and their bronchial wall thickness was not significantly different compared to PBS exposed *p21^-/-^* mice (Figure 2B). Therefore, p21 knockout alleviates the histological signs of chronic lung inflammation induced by chronic LPS exposure.

Matrix Metalloproteases (MMPs) are a family of proteinases that have the ability to degrade different extracellular matrix components [61]. MMP12, a member of the MMPs family, mediates alveolar destruction during emphysema and it promotes the pathogenesis of chronic bronchitis [62]. Therefore, we set to evaluate MMP12 expression following chronic LPS exposure in WT and *p21^-/-^* mice. As expected, WT mice that were exposed to LPS presented increased mRNA expression levels of *Mmp12* compared to the PBS exposed mice. However, p21 knockout decreased this effect, and *p21^-/-^* mice presented significantly lower levels of *Mmp12* then observed in the LPS exposed WT mice (Figure 2C). We then evaluated the accumulation of fibrosis in our model. Sirius Red staining, which stains ECM (Supplementary Figure 2A, 2B) and protein expression of COL1 (Supplementary Figure 2C) revealed no significant changes between PBS and LPS treated WT mice. Therefore, while *Mmp12* levels are affected by LPS exposure, no significant accumulation of fibrotic tissue was observed following this treatment.

Since chronic bronchitis causes damages to the lung in a manner that affects fitness [63], we set to examine whether chronic LPS exposure has an effect on the mice fitness. We subjected the mice to a treadmill assay, a behavioral test that measures the ability of mice to sustain running. The assay was performed at 5 time points, specifically, 3 days before the first LPS exposure (hereafter, baseline), 3, 5, 7 and 9 weeks in-parallel with LPS exposure. We observed that following 9 weeks of chronic LPS exposure, the maximal actual distance mice run until exhaustion was significantly shorter among LPS exposed WT mice compared to PBS exposed WT mice (Figure 2D). In contrast, the maximal actual distance that *p21^-/-^* mice run until exhaustion did not differ between PBS and LPS exposed mice. Moreover, the delta maximal actual distance that was calculated from the baseline point was significantly higher in the PBS exposed WT mice compared to the LPS exposed WT mice (Figure 2E), but did not differ between *p21^-/-^* mice exposed to either PBS or LPS. These results indicate that the LPS exposure regimen reduces the fitness of WT, but not *p21^-/-^* mice exposed to LPS at the end of the exposure period. Altogether, p21 knockout limits LPS-induced chronic bronchitis as manifested both at tissue pathology and organismal fitness.

### p21 promotes chronic inflammatory responses in the lung airways

**Figure 3:**
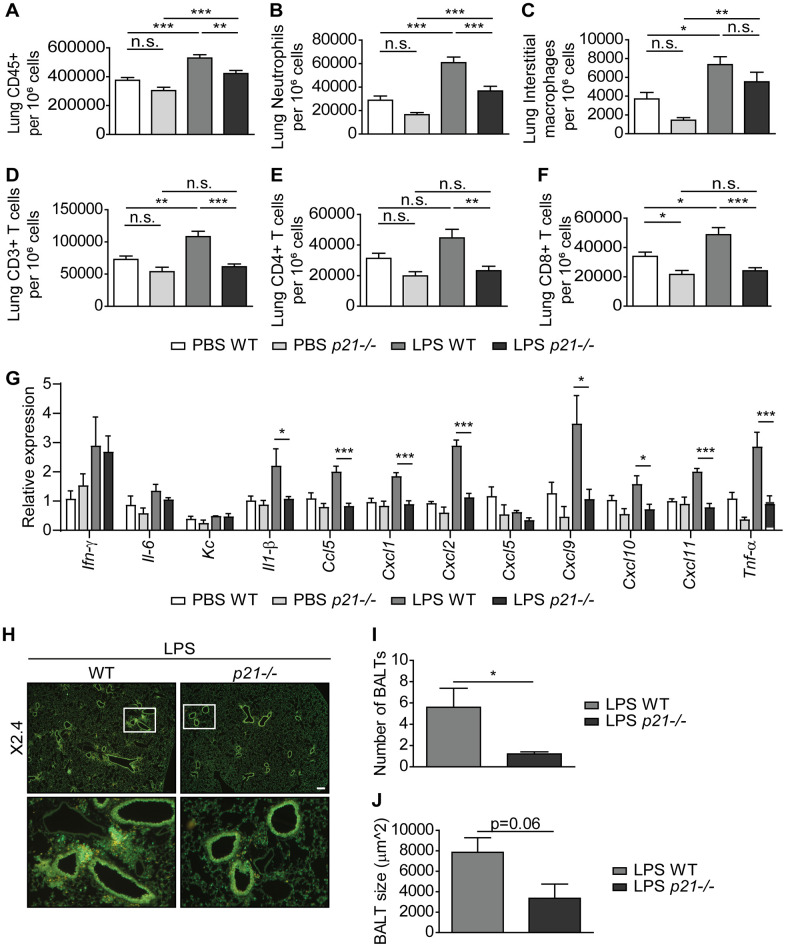
***p21^-/-^* decreases chronic inflammatory responses and iBALT formation caused by exposure to aerosolized LPS.** WT and *p21^-/-^* mice were exposed to either PBS or aerosolized LPS (0.5 mg/ml), 3 times a week for 10 weeks. (**A**–**F**) At 48 hours following the last LPS exposure, whole lungs were dissociated into single cell suspensions and analyzed by flow cytometry to determine: (**A**) numbers of immune cells (CD45+), (**B**) numbers of neutrophils (CD45+/Ly6G+/CD11b+), (**C**) numbers of interstitial macrophages (CD45+/CD11c+/SiglecF-/CD11b+/CD24+), (**D**) numbers of CD3+ T cells (CD45+/CD3+), (**E**) numbers of CD4+ T cells (CD45+/CD3+/CD4+), and (**F**) numbers of CD8+ T cells (CD45+/CD3+/CD8+). (**G**) mRNA expression levels of the indicated SASP factors in the mice lungs. (**H**) Representative images of lungs stained for CD3+ (red) and B220 (green) depict accumulation of iBALTs in LPS exposed mice. Scale bar represents 200μm. (**I**, **J**) Numbers (**I**) and sizes (**J**) of iBALTs in the lungs of mice exposed to aerosolized LPS. Data information: Data were analyzed using one-way ANOVA, *P<0.05. **<0.005. ***P<0.0005 (**A**–**G**), and by Student’s t-test, *p<0.05, **p<0.01, and ***p<0.005 (**I**, **J**). Data represent mean ±SEM (**A**–**F**, n=9-12; **G**, n=3-6; **H**–**J**; n=4-6 independent repeats).

A central hallmark of chronic lung inflammation is the recruitment and accumulation of immune cells within the lung parenchyma and the bronchoalveolar lavage (BAL) fluid [9, 64]. Therefore, to determine whether *p21^-/-^* affects the chronic inflammatory response in the lungs, we examined the cellular immune response in both the lung parenchyma and the BAL fluid of mice subjected to the chronic LPS regimen. The cellular immune response and infiltration of both innate and adaptive immune cells in the mice lung parenchyma following chronic LPS exposure was assessed by flow cytometry. This analysis revealed a significantly higher influx of CD45+ immune cells (Figure 3A) in the lungs of LPS exposed WT mice compared to the PBS exposed WT mice. By analysing the presence of specific components of the immune system, we revealed a significant accumulation of neutrophils, interstitial macrophages, CD3+, CD4+, and CD8+ T cells (Figure 3B–3F) following chronic LPS exposure. Remarkably, p21 knockout resulted in a significantly lower accumulation of all these immune cell subsets (Figure 3A–3F). The inflammatory response in the lung is accompanied by an elevation in inflammatory secreted components, such as cytokines and chemokines [65–67]. To examine whether p21 knockout altered the inflammatory response in LPS-exposed mice, we analyzed the mRNA expression levels of pro-inflammatory SASP components. We observed a significant increase in the expression of *Il-1β, Ccl5, Cxcl1*, *Cxcl2*, *Cxcl9, Cxcl10, Cxcl11* and *Tnf-α* in LPS exposed WT mice compared with PBS exposed mice, but not in *p21^-/-^* mice (Figure 3G). In both WT and *p21^-/-^* mice, however, the LPS-triggered increase in *Ifn-γ, Il-6*, *Kc* and *Cxcl5* remained the same.

To understand the inflammatory response in the lung parenchyma, we explored *in-situ* the location of the adaptive immune populations of T and B cells, which aggregate in the LPS-exposed mice lungs. These aggregations present histological features of inducible bronchus-associated lymphoid tissue (iBALT). Using immunofluorescence co-staining we detected the presence of iBALTs in the chronic LPS exposed mice lungs (Figure 3H). Notably, in *p21^-/-^* LPS exposed mice, the iBALTs number was 4-fold lower than in WT LPS exposed mice (Figure 3I). In addition, the iBALTs size was smaller in the *p21^-/-^* mice compared to the WT mice (Figure 3J, p=0.06). Since *p21^-/-^* mice presented a low number of iBALTs in comparison to WT mice, fewer iBALTs were measured, thus negatively affecting the ability to identify the difference between the genotypes. However, it is evident that *p21^-/-^* leads to a reduction in the number and the size of iBALTs, indicating lower level of immune cell accumulation in these mice.

**Figure 4:**
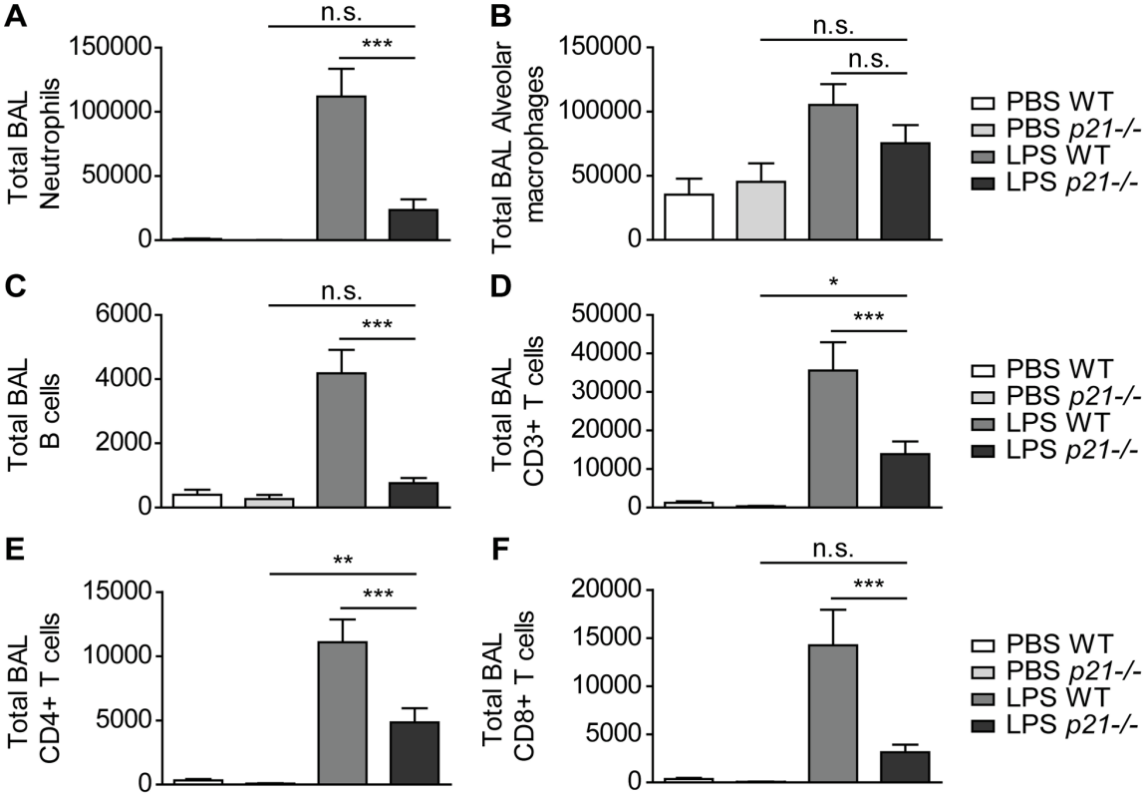
***p21^-/-^* protects lung airways from chronic inflammatory responses triggered by chronic LPS exposure.** WT and *p21^-/-^* mice were exposed to either PBS or aerosolized LPS (0.5 mg/ml), 3 times a week for 10 weeks. (**A**–**F**) At 48 hours following the last LPS exposure BAL fluid was collected and flow cytometry was used to determine: (**A**) numbers of neutrophils (CD45+/Ly6G+/CD11b+), (**B**) numbers of alveolar macrophages (CD45+/CD11c+/SiglecF+), (**C**) numbers of B cells (CD45+/B220+), (**D**) numbers of CD3+ T cells (CD45+/CD3+), (**E**) numbers of CD4+ T cells (CD45+/CD3+/CD4+), and (**F**) numbers of CD8+ T cells (CD45+/CD3+/CD8+). Data information: Data were analyzed using one-way ANOVA. ***P<0.0005. Data represent mean ±SEM (**A**–**F**, n=8-11 independent repeats).

Then, we evaluated the cellular immune response in the mice BAL fluid by flow cytometry. As in the lung parenchyma, we observed a significantly higher influx of neutrophils, B cells, CD3+, CD4+ and CD8+ T cells (Figure 4A, 4C–4F), accompanied by an elevated influx of alveolar macrophages (Figure 4B) in WT LPS exposed mice compared to the PBS exposed WT mice. In contrast, a 2-fold decrease on average in the influx of these immune cell populations was observed in *p21^-/-^* LPS exposed mice (Figure 4A–4F). It, therefore, appears that p21 knockout alleviates the accumulation of immune cells in lungs, including specifically in iBALTs, following chronic LPS exposure.

### Epithelial and endothelial cell populations mediate p21-dependent inflammatory responses following chronic LPS inhalation

**Figure 5:**
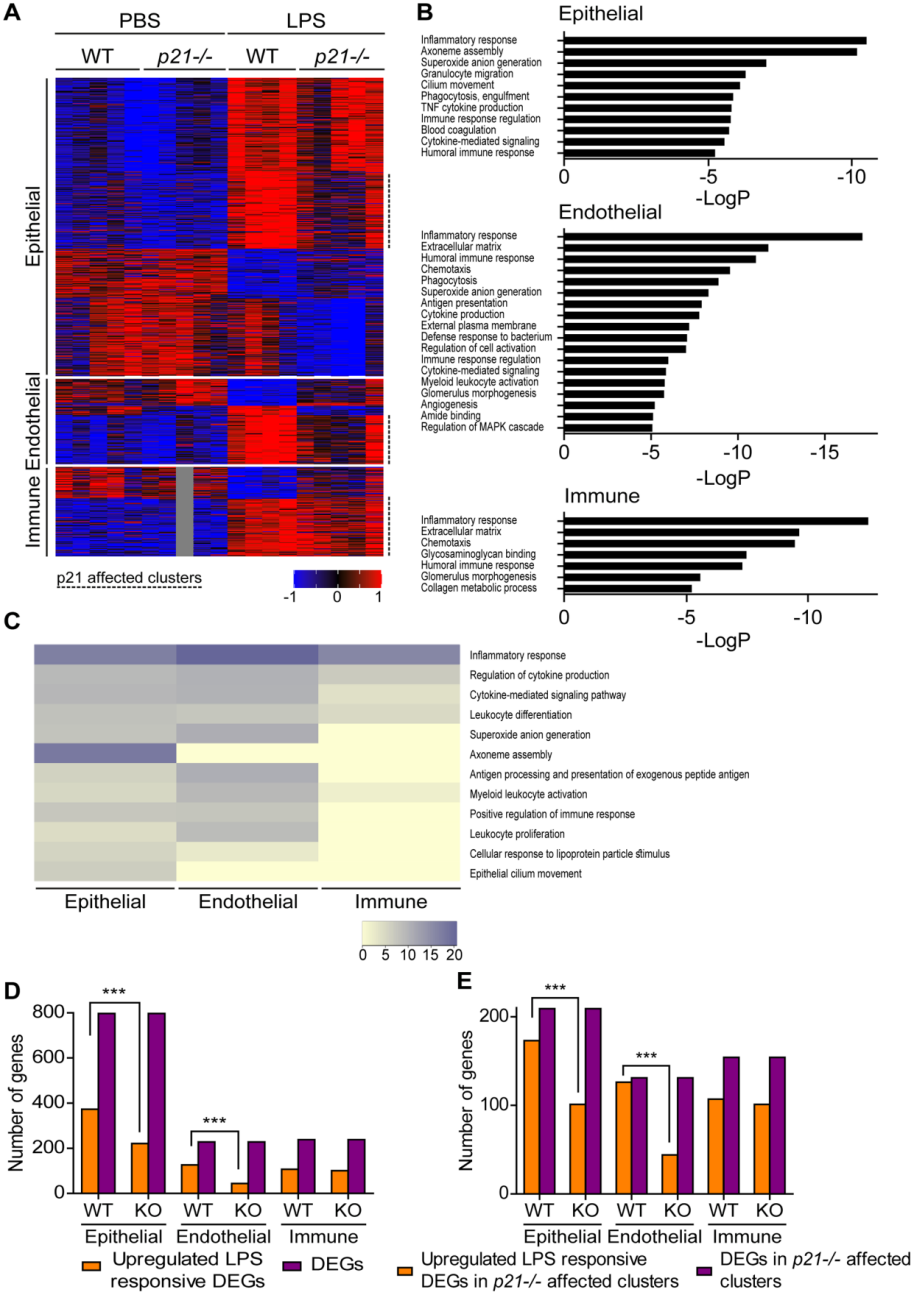
**Epithelial and endothelial cell populations mediate p21-dependent inflammatory responses following chronic LPS inhalation.** WT and *p21^-/-^* mice were exposed to either PBS or aerosolized LPS (0.5 mg/ml), 3 times a week for 10 weeks. At 48 hours following the last LPS exposure, whole lungs were dissected and dissociated into single cell suspensions and analyzed by flow cytometry and sorting for subsequent RNA sequencing. Data was analyzed as follows: (**A**) Hierarchical clustering heatmap of differentially expressed genes (DEGs) in epithelial, endothelial and immune cells populations in the mice lungs. One sample of the immune cells population, in the PBS *p21^-/-^* mice group, was excluded from the analysis. (**B**) Functional analysis of the DEGs in epithelial, endothelial and immune cells populations in the mice lungs. Abbreviations: antigen processing and presentation - antigen presentation, cytokine-mediated signaling pathway - cytokine-mediated signaling, external side of plasma membrane - external plasma membrane, glomerulus vasculature morphogenesis - glomerulus vasculature morphogenesis, positive regulation of immune response - immune response regulation, positive regulation of MAPK cascade - regulation of MAPK cascade, regulation of cytokine production - cytokine production, regulation of tumor necrosis factor superfamily cytokine production - TNF cytokine production. (**C**) Heatmap based on functional analysis of the upregulated DEGs in epithelial, endothelial and immune cells populations in the mice lungs. (**D**) Number of upregulated LPS responsive DEGs in epithelial, endothelial and immune cells populations in the mice lungs. (**E**) Number of upregulated LPS responsive DEGs in *p21^-/-^* affected clusters in epithelial, endothelial and immune cells populations in the mice lungs. Data information: Data were analyzed using Chi-squared test. ***P<0.0005. (**A**–**E**), n=4-5 independent repeats.

Tissue physiology and pathophysiology are dictated by the presence and interaction of different cell types. Of note, LPS treatment results in profound changes in inflammation and immune cells recruitment to the lungs. To better understand the role of p21 in different cell types in the inflammatory response during chronic lung inflammation, we performed a transcriptome analysis for the lung’s three most abundant cell populations, namely, epithelial, endothelial and immune cells. Due to the absence of fibrosis in this experimental system (Supplementary Figure 2A–2C), the amount of fibroblasts in the lung is relatively small. Therefore, this cell population was not evaluated. The cells were FACS-sorted from WT and *p21^-/-^* mice lungs and subjected to mRNA sequencing. We analyzed the data for each cell type separately and identified the differentially-expressed genes (DEGs) affected by LPS treatment and/or p21 knockout. We performed three distinct comparisons: LPS WT vs. PBS WT, LPS *p21^-/-^* vs. PBS *p21^-/-^* and LPS *p21^-/-^* vs. LPS WT. Out of these comparisons, we selected the DEGs that passed the threshold of |log2FoldChange|≥1, average mean normalized reads |≥5 and adjusted p value≤0.05 in at least one of the comparisons. 13754, 12947 and 12806 genes were identified in epithelial, endothelial and immune cells, respectively. Out of the identified genes, 797, 228 and 238 DEGs were identified in epithelial, endothelial and immune cells, respectively. We performed partitioning clustering for each cell type and presented the data as a heatmap (Figure 5A). For each cell population we examined the clusters and observed that following chronic LPS exposure WT mice presented clusters of DEGs that were upregulated in epithelial (5 clusters), endothelial (3 clusters) and immune cell (2 clusters) populations, compared to PBS exposed mice. However, p21 knockout resulted in a general downregulation in the expression of DEGs in the p21-affected clusters. Then, we performed a functional analysis of the genes of the p21-affected clusters to uncover the enriched Gene Ontology (GO) terms of each specific cell population (Figure 5B). The “inflammatory response” term was enriched in all cell populations, reflecting the severe response to chronic LPS exposure. Surprisingly, however, the epithelial and endothelial cell populations showed enrichment in 8 and 11 GO terms, respectively, related to inflammatory pathways, while the immune cells were enriched only in 3 such GO terms (Figure 5B).

To reveal the possible functional impact of the upregulated DEGs, we performed a functional analysis to compare the enrichment of the most significant GO terms between epithelial, endothelial and immune cells populations. We observed that both epithelial and endothelial cell populations presented enrichment of inflammatory response related terms such as “superoxide anion generation”, “antigen processing and presentation of exogenous peptide antigen”, “positive regulation of immune response” and “leukocyte proliferation”, which, surprisingly, were not enriched in the immune cells population (Figure 5C). Interestingly, we observed that the terms “axoneme assembly” and “epithelial cilium movement”, which are functionally related to epithelia, were enriched only in the epithelial cell population.

To further examine which genes are affected by p21 following LPS treatment in each cell population, we compared the upregulated DEGs following LPS treatment in both WT (LPS WT vs. PBS WT) and *p21^-/-^* (LPS *p21^-/-^* vs. PBS *p21^-/-^*) mice. First, a KEGG pathway analysis in epithelial, endothelial and immune cells revealed no differences in gene expression when comparing between PBS WT and PBS *p21^-/-^* mice. In contrast, we observed that chronic LPS exposure resulted in a significant upregulation of DEGs in WT mice compared with *p21^-/-^* mice, in the epithelial (p<0.001) and endothelial (p<0.001) cell populations, but not in the immune cells population (p=0.543) (Figure 5D). Furthermore, a KEGG pathway analysis revealed that chronic LPS exposure resulted in changes in the inflammatory response, the p53 signaling pathway and cellular senescence pathways in WT mice compared with *p21^-/-^* mice in endothelial cells (Supplementary Figure 3A).

Then, in order to determine whether the differences observed in the epithelial and endothelial cell populations following chronic LPS exposure are p21-dependent, we compared the amount of upregulated DEGs following LPS treatment specifically in the p21 affected clusters in both WT (LPS WT vs. PBS WT) and *p21^-/-^* (LPS *p21^-/-^* vs. PBS *p21^-/-^*) mice. We observed that chronic LPS exposure resulted in a significant upregulation of DEGs in the p21 affected clusters in WT mice compared with *p21^-/-^* mice, in the epithelial (p<0.001) and endothelial (p<0.001) cell populations, but not in the immune cells population (p=0.644) (Figure 5E). Therefore, the effect of p21 knockout is prominent in epithelial and endothelial but not in immune cells. Overall, this analysis suggests that resident epithelial and endothelial cells in the lungs, but not immune cells, play a significant role in mediating the p21-dependent regulation of inflammatory response following chronic LPS exposure.

## DISCUSSION

The long-term persistence of senescent cells is deleterious and can promote the development of age-related pathologies [28, 29, 68]. Here, we show that senescent cells accumulate during chronic bronchitis in mice, and p21 knockout decreases their presence. We observed a significant decrease in the main characteristics of chronic bronchitis in p21 knockout, compared to WT mice. The main characteristics affected were the influx of immune cells into the BAL fluid and lung parenchyma, inflammatory cytokines in the broncho-alveolar spaces, and the pathological manifestations of chronic bronchitis, including iBALTs. These results show that p21 knockout reduces the long-term immunological response to LPS. However, the signs of inflammation, including CD3+ and CD4+ T cells in the BAL fluid and the neutrophils and interstitial macrophages in the lung parenchyma, were still present in LPS treated p21 knockout mice. Furthermore, our study illustrates that resident epithelial and endothelial cells in the lungs, but not immune cells, play a significant role in mediating the p21-dependent inflammatory response during chronic bronchitis. These findings suggest that p21 is a crucial regulator of chronic airway inflammation and lung destruction and a significant driver of chronic bronchitis. These effects are mediated through epithelial and endothelial cells.

The increase in p21 levels maintains the viability of senescent cells following the stress they might undergo in pathological conditions [60]. Senescent cells expressing p21 contribute to the progression of lung pathologies, such as lung fibrosis and chronic lung inflammation [54, 68–72]. Moreover, p21 deficiency reduces senescent cells accumulation and protects mice from liver fibrosis [60], renal fibrosis [73], osteopenia [74] and chronic pancreatitis [75]. Emerging evidence suggests that various types of cells undergo senescence and upregulate p21 in lungs affected by chronic bronchitis, including in alveolar cells, bronchial epithelial cells, fibroblasts, smooth muscle cells, endothelial cells, and leukocytes [72, 76–78]. These cells can contribute to the inflammatory response following chronic LPS exposure through secretion of SASP in an autocrine and paracrine manner. However, the specific contribution of each cell type population to the p21-dependent inflammatory response has yet been examined.

Senescent cells of different cell types might affect the progression of chronic bronchitis differently. Senescence of both adaptive and innate immune cells (immunosenescence) may result in impaired immune responses and aggravate lung inflammation [79–81]. However, here we showed that the p21-dependent inflammatory response following chronic LPS exposure is not mediated by the immune cells, but rather by resident epithelial and endothelial cells in the lungs. Indeed, epithelial and endothelial cells accumulate in the lungs during chronic bronchitis [76, 82]. These cells may contribute differently to the progression of chronic bronchitis pathology. Accumulation of p21-positive senescent airway epithelial cells during chronic bronchitis results in impaired lung epithelial regeneration, in addition to accelerated p38 MAPK-dependent airway inflammation [83]. Deleting p21 in epithelial cells also attenuates DNA damage, inflammatory responses, and oxidative stress induced by chronic bronchitis [54, 84]. p21 expression in senescent endothelial cells contributes to the inflammatory response by secretion of SASP [77, 85], while promoting immune cells adhesion by expression of ICAM-1 [77]. Overall, p21 in epithelial and endothelial cells potentiates the pro-inflammatory response in chronic bronchitis following repeated injury by various mechanisms. Ablation of p21 disrupts the accumulation of senescent cells and alleviates chronic lung inflammation.

Elimination of senescent cells may allow for better recovery of tissues from damage. Indeed, senescent cells elimination via transgenic techniques improves aging-associated phenotypes [31, 38, 86]. Interestingly, p21 knockout supports tissue regeneration and limits aging phenotypes in several mouse models. For example, p21 prolongs the lifespan of telomerase deficient mice by rescuing the proliferation of intestinal progenitor cells and improving the repopulation capacity of hematopoietic stem cells [87]. Additionally, p21 deficiency supports tissue regeneration, including regeneration of articular cartilage [88, 89], bone regeneration following injury [90] and skin wound healing [91]. Lastly, p21 attenuates the phenotype of aged BubR1 hypomorphic progeroid mice [92]. Therefore, we suggest that p21-dependent elimination of senescent cells may limit the damage induced by the pro-inflammatory presence of senescent cells, but also promote tissue regeneration. Therefore, inhibition of p21 represents a promising strategy for limiting age-related inflammatory disorders in general and obstructive lung diseases in particular.

## MATERIALS AND METHODS

### Contact for reagent and resource sharing

Further information and requests for reagents may be directed to, and will be fulfilled by the corresponding author, Dr. Valery Krizhanovsky (valery.krizhanovsky@weizmann.ac.il). All the key resources used in this study are summarized in the Key resources table.

### Methods and protocols

### Mice

Female C57BL/6J mice were purchased from Harlan Laboratories. *p21^-/-^* mice [B6.129S6(Cg)-Cdkn1a<tm1Led>/J] mice were obtained from the Jackson Laboratory (#016565).

### Chronic lung inflammation induction

Chronic LPS exposure regime was performed to age-matched C57BL/6J and *p21^−/−^* mice that were exposed to PBS or PBS containing *Escherichia coli* LPS (L2630, Sigma-Aldrich) aerosol, as previously described by us [26]. Following 48 hours from the last exposure all mice were harvested. BAL fluid and right lung lobes were subjected to fluorescence-activated cell sorting (FACS) analysis. Left lung lobes were taken for cell sorting, RNA and protein extraction, fixation using 4% paraformaldehyde (PFA) for immunohistology or embedded in OCT solution for cryosectioning and SA-β-gal stains.

### Single-cell lung homogenate preparation, FACS analysis and sorting

The lungs of euthanized mice were removed, washed in RPMI medium (11875-093, Thermo Fisher Scientific), minced, and incubated at 37C for 45min in RPMI medium containing 1mg/ml collagenase type 4 (LS004189, Worthington), and 0.02mg/ml DNaseI (04536282001, Roche). Lung cell suspensions were pushed through a 100-μm cell strainer and spun, and red blood cells were lysed with red blood cell lysis buffer (R7757, Sigma-Aldrich). Cells from whole lungs were collected, as well as BAL fluid, washed twice with FACS buffer, and immunolabeled with antibodies against B220 (PerCP/Cy5.5, #103236), CD4 (APC, #100412), CD8a (FITC, #100706), CD11b (PerCP/Cy5.5, #101228), CD11c (APC, #117310), CD24 (PE-CY7, #101822), CD45 (PB, #103126 or FITC, #103108), CD103 (PE, #121406), Ly6G (APC, #127614), MHC-II (APC-CY7, #107627), NKp46 (PE, #137604), TCR-b (PE, #109208) (all from BioLegend) and Siglec-F (PB, #562681, BD Biosciences). The cells were run in a LSR II Flow Cytometer (BD Biosciences) and analyzed using the FlowJo v10 software (BD Biosciences). The gating strategy for the immune subsets was performed as follows: neutrophils (CD45+/Ly6G+/CD11b+), NK cells (CD45+/NKp46+), CD3 (CD45+/TCRb+), CD4 (CD45+/TCRb+/CD4+), CD8 (CD45+/TCRb+/CD8+), B cells (CD45+/B220+), alveolar macrophages (CD45+/CD11c+/SiglecF+) and interstitial macrophages (CD45+/MHCII+, CD11c+, Siglec-F-, CD11b+, CD24-).

For sorting, the lungs of euthanized mice were perfused with cold PBS and then removed. The lungs were then washed in DMEM/F12 media (11330-032, Invitrogen), minced, and incubated at 37C for 20min in DMEM/F12 medium containing 3U/ml elastase (LS002279, Worthington), and 0.33U/ml DNaseI (04536282001, Roche). Lung cell suspensions were then washed with DMEM/F12 supplemented with 100units/ml of penicillin, 100mg/ml of streptomycin (03-031-1B, Biological Industries) and 10% fetal bovine serum (FBS) (Thermo Fisher Scientific), pushed through a 100-um cell strainer and spun. Red blood cells were lysed with ACK lysis buffer (A1049201, Gibco). The cells were then centrifuged, re-suspended in FACS buffer and immunolabeled with antibodies against CD31 (BV605, #102427), CD45 (PE, #103106), EpCam (Alexa Fluor 488, #118210) (all from BioLegend) and TER-119 (eFluor450, #48-5921-82, eBioscience). Cells were stained with SytoxBlue (S34857, Thermo Fisher Scientific) and were sorted using FACS Aria Fusion (BD Biosciences) directly into RLT buffer (74104, QIAGEN) for qPCR or Lysis/Binding buffer (61012, Thermo Fisher Scientific) for subsequent RNA sequencing. The gating strategy for the immune subsets was performed as follows: epithelial cells (CD45-/CD31-/EpCam+), endothelial cells (CD45-/CD31+/EpCam-) and immune cells (CD45+/CD31-/EpCam-).

### Quantitative RT-PCR

Total RNA from mice lungs was extracted using NucleoSpin RNA Mini kit (740955.50, Macherey-Nagel). cDNA was produced using random hexamers (N8080127, Thermo-Fisher). The cDNA samples were amplified using Platinum SYBR Green qPCR SuperMix (11744-500, Life Technologies) in a StepOnePlus Real-Time PCR System (Applied Biosystems). Relative expression was normalized using the expression levels of GAPDH. Primer sequences can be found in Supplementary Table 1.

### Immunoblotting

Cell lysates (15–30 mg of protein) were resolved by 12.5% SDS–PAGE and transferred onto ImmobilonP membranes (IPVH00010, Millipore). After blocking of the membranes with 5% bovine serum albumin (BSA) in TBST (Tris-buffered saline with 0.01% Tween-20) for 1 hour, they were probed with antibodies against p15 (ab53035), p16 (ab108349) (both from Abcam), cleaved-caspase 3 (#9661, Cell Signaling Technology), GAPDH (MAB374, Millipore), collagen-1 (600-401-103-0.5, Rockland Immunochemicals), p21 (sc-6246, Santa Cruz) and p53 (mix of DO-1 and PAb1801, kindly provided by M. Oren, Weizmann Institute of Science). The blots were developed using either SuperSignal West Pico PLUS chemiluminescent substrate (#34579) or SuperSignal West Femto maximum sensitivity substrate (#34095) (both from Thermo Fisher Scientific). The blots were analyzed using the Image Lab software (Bio-Rad Laboratories).

### Histological analysis

Immunofluorescence (IF) was performed on 4-μm paraffin sections and immunohistochemistry (IHC) was performed on 2-μm paraffin sections, according to standard procedures. Primary antibodies recognizing CD3 (1:50, MCA500G, Bio-Rad), CD45R (#14-0452-82, eBioscience, 1:50), cleaved caspase 3 (1:50, #9661, Cell Signaling Technology) and p21 (1:50, 556431, BD Biosciences) were applied overnight at 4C. Cleaved caspase 3 and p21 sections were developed using DAB (SK-4100, Vector Laboratories) followed by hematoxylin counterstaining. CD3 and CD45R sections were incubated with Cy3 anti-rabbit antibody (111-165-144, Jackson ImmunoResearch) for 90min in a humidity chamber. Sections were counterstained by DAPI (D9542, Sigma-Aldrich) and then mounted using Fluormount (0100-01, Southern Biotech). Cleaved caspase 3 and p21 stained sections were examined and photographed with a bright field microscope (Olympus IX81) and images were visualized using the CellP software (Diagnostic Instruments). CD3 and CD45R stained sections were visualized and photographed with a fluorescence microscope (Eclipse Ni-U; Nikon) equipped with Plan Fluor objectives (20x; 40x; 60x) connected to a color camera (DS-Ri1, Nikon) microscope. Images were analyzed using the Image Pro+ software (Nikon).

Paraffin-embedded tissue sections (4-μm) were stained with hematoxylin–eosin (H&E) for routine examination. From these stained slides bronchial epithelial cell wall width were calculated. 10 random points were chosen and the average of the cell wall width was measured. The sections were also stained for with Sirius Red for visualization of fibrotic deposition. The sections were examined and photographed with a bright-field microscope (Olympus). These images were then quantified using the NIH ImageJ software (http://rsb.info.nih.gov/ij/).

For SA-β-gal staining, 14-μm cryosections of OCT embedded mouse lungs were fixed in 0.5% glutaraldehyde (50-261-96, Electron Microscopy Sciences) for 15min, washed with PBS supplemented with 1mM MgCl_2_ in PBS at pH 5.5, and incubated for 6 hours in X-Gal staining solution (1mg/mL X-Gal (1758-0300, Inalco Pharmaceuticals), 5mM potassium ferrocyanide (104973, Merck Millipore), 5mM potassium ferricyanide (P3289, Sigma-Aldrich) and 1mM MgCl_2_ in PBS at pH 5.5). Sections were counterstained with Nuclear Fast Red (N3020, Sigma-Aldrich), dehydrated and mounted with Eukitt mounting solution (03989, Sigma-Aldrich). Sections were visualized using an Olympus microscope, and images were analyzed using the CellP software (Diagnostic Instruments).

### Treadmill assay

Mice were tested before and during chronic LPS exposure. The assay was performed on a treadmill (Panlab Mouse 5-Lane Treadmill; model#: 760309; HARVARD APPARATUS, USA) at 5 time points: baseline point (3 days before the first LPS exposure) and every other week from week 3 until week 9. Training protocol was described [93] and modified as follows: mice run on the treadmill with no incline, starting at a speed of 15 meters/min for 10min. Afterwards, the speed was increased gradually, over 10min, to a final speed of 35 meters/min. Finally, mice run at a speed of 35 meters/min for another 10min. The maximal actual distance was determined from the starting point until exhaustion (defined as 2 consecutive 30sec intervals in which the mouse steps off the lane at least 5 times).

### MARS-Seq sequencing and data analysis

Libraries were prepared from pooled samples of the same cell type (10000 cells per sample) according to a bulk variation of MARS-Seq [94] and were sequenced on Illumina NextSeq500 (Illumina). Data was analyzed using a User-friendly Transcriptome Analysis Pipeline (UTAP) [95]. Only reads with unique mapping to the 3’ of RefSeq annotated genes (mm10, NCBI *Mus musculus* Annotation Release 109) were analyzed. Gene expression was calculated and normalized using DESeq2 with batch correction. A gene was defined differentially-expressed (DE) according to the following thresholds of adjusted p value ≤0.05, average normalized reads one of all samples ≥5, and absolute value of fold-change ≥2. We selected differentially-expressed genes (DEGs) from the following comparisons: LPS WT- PBS WT, LPS *p21^−/−^*- PBS *p21^−/−^* and LPS *p21^−/−^*-LPS WT. We performed partitioning clustering using the k-means algorithm (Euclidian distance) using Partek Genomics Suite® software (version 7.0). Gene ontology term enrichment analysis was performed using the Metascape software [96].

### Cytoscape pathways analysis

Pathway analysis was performed using Cytoscape [97] and STRING application [98] for exploring protein-protein interactions and Enrichment Map application to postulate pathways. Differentially expressed genes having adjusted p-value≤0.05 and Log2 fold change≥0.5 were used as input to project differentially expressed pathways in each comparison. Pathways having FDR value smaller than 0.05 are displayed.

### Statistical analysis

Data are presented as mean ±SEM. Statistical significance was performed by the Prism software (GraphPad) and determined using Student’s t-test for pairwise comparisons or one-way ANOVA for comparing multiple groups, as well as by the R software (version 4.0.3) to determine chi-squared test for comparing the number of DEGs. Mann Whitney u test was performed in order to test the statistical significance of the physical activity and fitness of the mice. A P-value of < 0.05 was considered significant.

### Data availability statement

The data that support the findings of this study is available from the corresponding author, V.K., on reasonable request.

## Supplementary Material

Supplementary Figures

Supplementary Table 1
