# Supplementary material for: p21 facilitates chronic lung inflammation via epithelial and endothelial cells
Source: Aging (Albany NY). 2023 Mar 30;15(7):2395–417. doi: 10.18632/aging.204622 (PMC10120903; doi:10.18632/aging.204622)
Supplement: Supplementary Figures [file aging-15-204622-s001.pdf]

SUPPLEMENTARY FIGURES

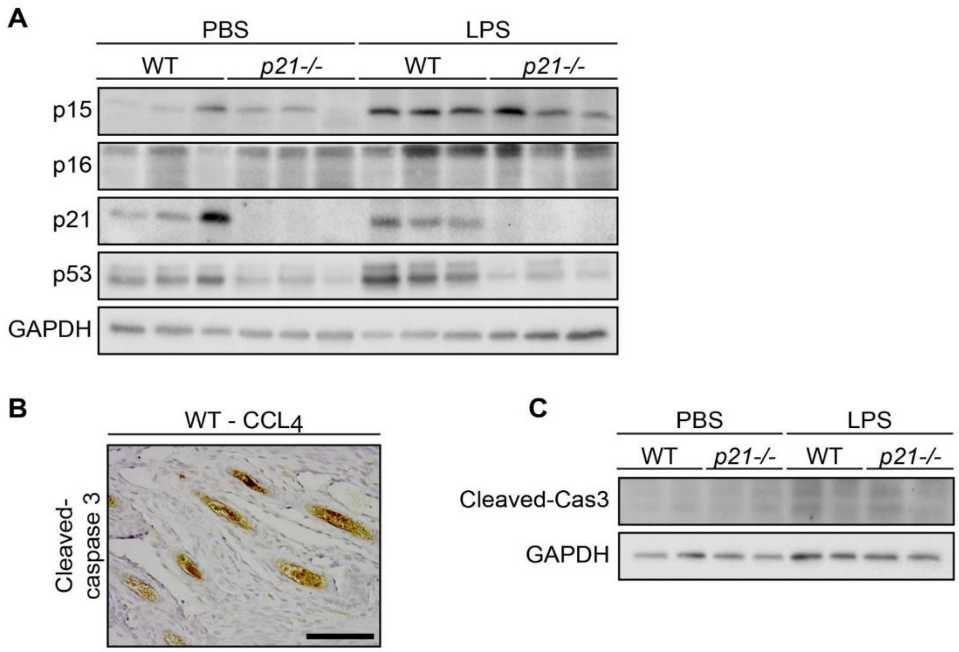

Supplementary Figure 1. Evaluation of senescence and apoptosis markers following chronic LPS exposure. Related to figure 1. WT and *p21*$^{-/-}$ mice were exposed to either PBS or aerosolized LPS (0.5 mg/ml), 3 times a week for 10 weeks. At 48 hours following the last LPS exposure, the lungs were harvested and frozen. Alternatively, lungs were harvested, fixed, and analyzed for markers of senescence. (**A**) Western blot analysis of senescence-associated proteins p15, p16, p21 and p53 in the mice lungs. (**B**) Immunohistochemistry (IHC) of liver section from WT mouse treated with CCl_4 for cleaved caspase 3. Scale bar represents 200µm. (**C**) Western blot analysis of apoptosis marker cleaved caspase 3 in the mice lungs. Data information: (**A**) n=3; (**B**) n=3; (**C**) n=2 independent repeats.

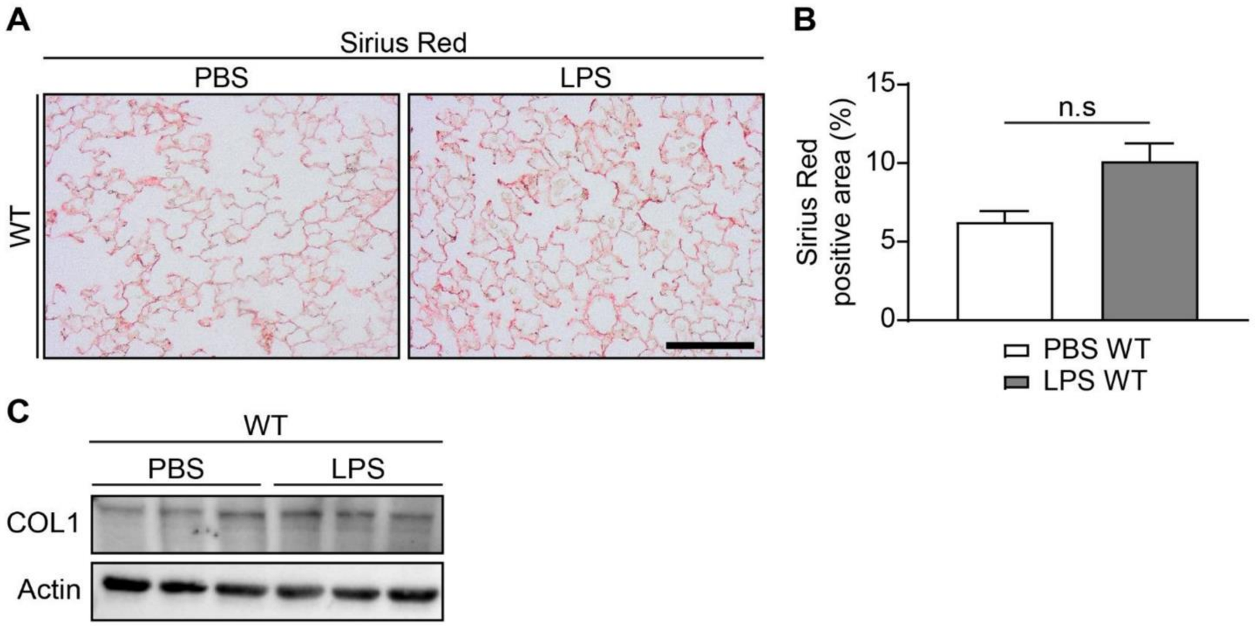

Supplementary Figure 2. Chronic LPS exposure does not lead to accumulation of fibrotic tissue. Related to Figure 2. WT and *p21*$^{-/-}$ mice were exposed to either PBS or aerosolized LPS (0.5 mg/ml), 3 times a week for 10 weeks. At 48 hours following the last LPS exposure, the lungs were harvested and frozen. Alternatively, lungs were harvested, fixed, and analyzed for fibrosis. (**A**) Lung sections were stained for Sirius Red. Scale bar: 200μM. (**B**) Quantification of Sirius Red staining presented in (**A**). (**C**) Western blot analysis of collagen-1 (COL1) in the mice lungs. Data information: Data were analyzed using Student's t-test, *p<0.05, **p<0.01, and ***p<0.005. Data represent mean ±SEM (**A**, **B**, n=3; **C**, n=3 independent repeats).

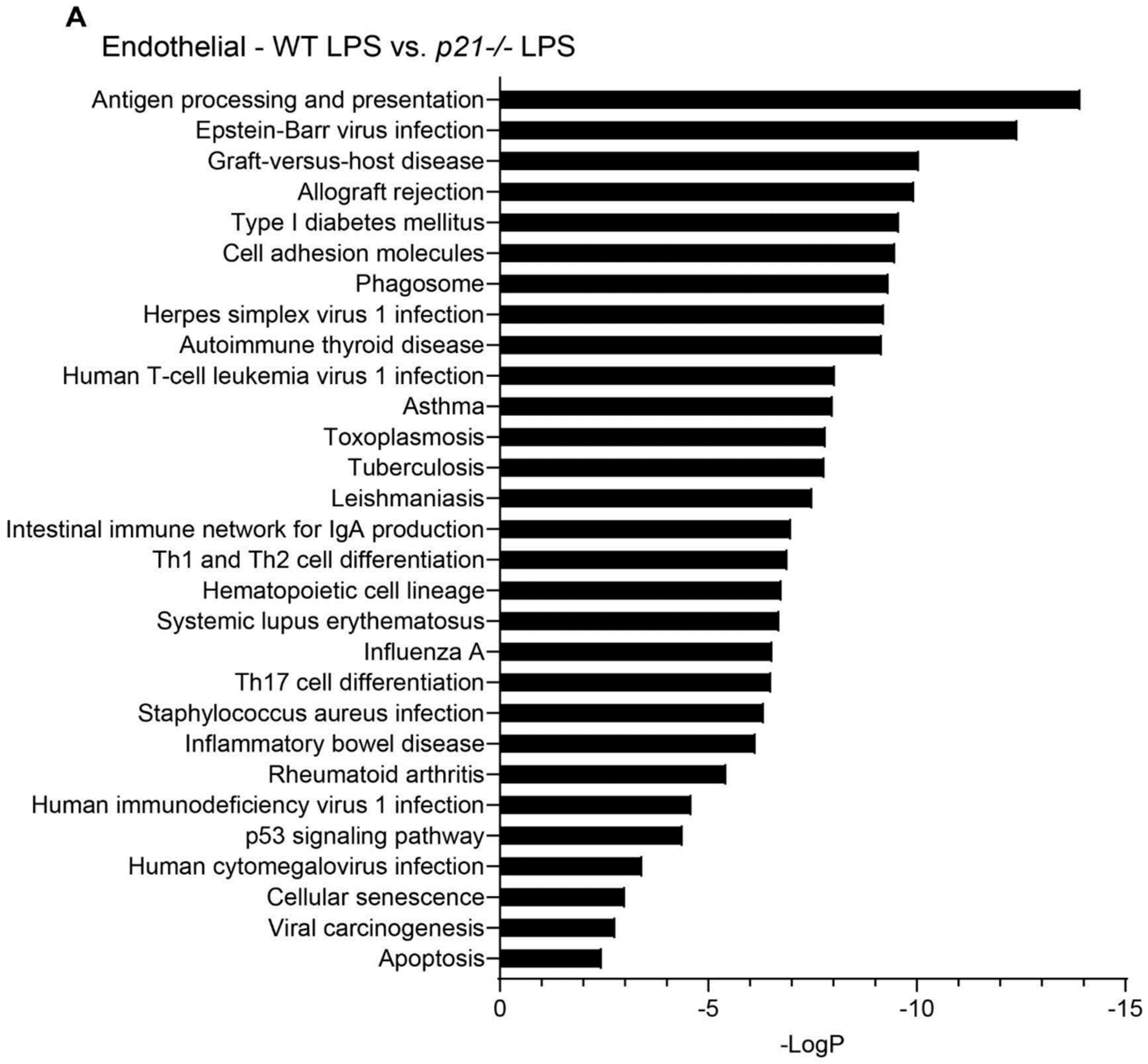

Supplementary Figure 3. KEGG pathway analysis in endothelial cells following chronic LPS exposure. Related to figure 5. WT and *p21*$^{-/-}$ mice were exposed to either PBS or aerosolized LPS (0.5 mg/ml), 3 times a week for 10 weeks. At 48 hours following 5 the last LPS exposure, whole lungs were dissected and dissociated into single cell suspensions and analyzed by flow cytometry and sorting for subsequent RNA sequencing. (**A**) KEGG pathway analysis of differentially expressed cellular pathways in endothelial cells population in the lungs of WT and *p21*$^{-/-}$ mice following chronic LPS exposure. Data information: (**A**) n=4-5 independent repeats.
