# Supplementary material for: p21 facilitates chronic lung inflammation via epithelial and endothelial cells
Source: Aging (Albany NY). 2023 Mar 30;15(7):2395–417. doi: 10.18632/aging.204622 (PMC10120903; doi:10.18632/aging.204622)
Supplement: Supplementary Table 1 [file aging-15-204622-s002.pdf]

SUPPLEMENTARY TABLE

Supplementary Table 1. List of qPCR mouse primers.

Primer	Forward sequence	Reverse sequence
GAPDH	5'-TGCACCACCAACTGCTTAGC-3'	5'-GGCATGGACTGTGGTCATGAG-3'
Ccl5	5′-GCCCTCACCATCATCCTCAC-3′	5′-ATCCCATTTTCCCAGGACC-3′
Cxcl1	5'-GGCGCCTATCGCCAATG-3'	5'-CTGGATGTTCTTGAGGTGAATCC-3'
Cxcl2	5'-GCCCAGACAGAAGTCATTGCC-3'	5'-CTCCTCCTTTCCAGGTCAGTTA-3'
Cxcl5	5'-GTTCCATCTCGCCATTCATGC-3'	5'-GCGGCTATGACTGAGGAAGG-3'
Cxcl9	5'-TCTTCCTGGAGCAGTGTGG-3'	5'-TCCGGATCTAGGCAGGTTT-3'
Cxcl10	5'-CCATCAGCACCATGAACC-3'	5'-TCCGGATTCAGACATCTC-3'
Cxcl11	5'-GCTCAAGGCTTCTTATGTTCAA-3'	5'-CTTTGTCGCAGCCGTTACTCG-3'
Ifn-γ	5'-CATGGCTGTTTCTGGCTGTTACTG-3'	5'-GTTGCTGATGGCCTGATTGTCTTT-3'
Il1-β	5'-GGAGAACCAAGCAACGACAAAATA-3'	5'-TGGGGAACTCTGCAGACTCAAAC-3'
Il-6	5'-AGACAAAGCCAGAGTCCTTC-3'	5'-TGCCGAGTAGATCTCAAAGT-3'
Kc	5'-AAGAATGGTCGCGAGGCTTG-3'	5'-TGCCATCAGAGCAGTCTGTC-3'
Mmp12	5'-GGAGCTCACGGAGACTTCAACT-3'	5'-CCTTGAATACCAGGTCCAGGATA-3'
p15	5'-CCACCCTTACCAGACCTGTG-3'	5'-AGGCGTCACACACATCCAG-3'
p16	5′-TTGGGCGGGCACTGAATCTC-3′	5′-AGTCTGTCTGCAGCGGACTC-3′
p21	5′-GACAAGAGGCCCAGTACTTC-3′	5′-GCTTGGAGTGATAGAAATCTGTC-3′
Tnf-α	5'-CCACGCTCTTCTGTCTACTG-3'	5'-GATGAGAGGGAGGCCATTTG-3'
